
JOURNAL INFORMATION
==============================
NLM Title Abbreviation: Innov Aging
Journal ID: Innov Aging
NLM Journal ID: 101703706
Journal ID: innovateage

Innovation in Aging

EISSN: 2399-5300
Publisher: Oxford University Press

ARTICLE INFORMATION
==============================
PMCID: PMC8681898
DOI: 10.1093/geroni/igab046.755
Article ID: igab046.755
Article version: 1
Subjects: Abstracts, Session 2245 (Symposium), AcademicSubjects/SOC02600

Insights Into Hippocampal Biology from Youth-Associated Plasma Factors

Castellano Joseph
Icahn School of Medicine at Mount Sinai, New York , New York , United States
Publication date: 2021
Electronic publication date: 2021 Dec 17
Volume: 5
Issue: Suppl 1
Issue title: Program Abstracts from The GSA 2021 Annual Scientific Meeting, “Disruption to Transformation: Aging in the “New Normal””
First page: 196
Last page: 197
Copyright: © The Author(s) 2021. Published by Oxford University Press on behalf of The Gerontological Society of America.
Copyright year: 2021
License: This is an Open Access article distributed under the terms of the Creative Commons Attribution License (http://creativecommons.org/licenses/by/4.0/), which permits unrestricted reuse, distribution, and reproduction in any medium, provided the original work is properly cited.
License URL: https://creativecommons.org/licenses/by/4.0/

Page count: 2

==============================

### INSIGHTS INTO HIPPOCAMPAL BIOLOGY FROM YOUTH-ASSOCIATED PLASMA FACTORS

Joseph Castellano, *Icahn School of Medicine at Mount Sinai, New York, New York, United States*
